# Ballistic and Collisional Flow Contributions to Anti-Fourier Heat Transfer in Rarefied Cavity Flow

**DOI:** 10.1038/s41598-018-31827-2

**Published:** 2018-09-10

**Authors:** Hassan Akhlaghi, Ehsan Roohi, Stefan Stefanov

**Affiliations:** 1 Department of Aerospace Engineering, Sharif University of Technology, 1458889694 Tehran, Iran; 2 High Performance Computing (HPC) Laboratory, Department of Mechanical Engineering, Ferdowsi University of Mashhad, P.O. Box, 91775-1111 Mashhad, Iran; 3 Institute of Mechanics, Bulgarian Academy of Science, Acad. G. Bontchev Str., 1113 Sofia, Bulgaria

## Abstract

This paper investigates anti-Fourier heat transfer phenomenon in a rarefied gas confined within a lid-driven cavity using a novel flow decomposition technique in the direct simulation Monte Carlo (DSMC) method proposed by Stefanov and co-workers. An isothermal cavity with different degrees of flow rarefaction from near continuum to mid transition regimes was considered to investigate cold-to-hot heat transfer from ballistic/collision flow decomposition viewpoint. A new cold-to-hot heat transfer indicator in the form of a scalar product of normalized heat flow vector and normalized temperature gradient vector has been introduced for the overall, ballistic and collision parts of these vectors. Using the new indicator, contributions of ballistic and collision flow parts to temperature and heat flux components was investigated with a specific emphasis on the cold-to-hot heat transfer phenomenon. We demonstrated that both ballistic and collision flow parts contribute to the occurrence of cold-to-hot heat transfer. However, it was found out that considered separately both ballistic and collision parts of heat transfer, when related to corresponding ballistic and collision temperature fields, they are ever hot-to-cold for all degrees of flow rarefaction. Thus, cold-to-hot heat transfer is a result of a subtle interplay between ballistic and collision parts in the slip and transition Knudsen regimes.

## Introduction

Recently, the anti-Fourier (also known as cold-to-hot or counter-gradient) heat transfer phenomenon has been reported in rarefied gas flows^1–6^ based on molecular simulations of gas flow using Direct Simulation Monte Carlo (DSMC) method. John *et al*.^1^ investigated the role of expansion cooling and viscous dissipation on the heat transfer mechanism in a lid-driven cavity under nonequilibrium flow conditions. They observed a counter-gradient heat transfer in the slip-flow regime using DSMC simulations which cannot be predicted by the classical Navier-Stokes-Fourier (NSF) equations. Mohammadzadeh *et al*.^2^ investigated thermal characteristics of micro- and nanocavity flow in details. They demonstrated that the existence of unconventional cold-to-hot heat transfer is strongly dependent on the Reynolds number and it vanishes for large Reynolds/Mach number flows. Using molecular entropy analysis, they showed that the cold-to-hot heat transfer in the cavity is well in accordance with the second law of thermodynamics and takes place in the direction of increasing entropy.

Rana *et al*.^3^ showed that contrary to the NSF equations, the DSMC and regularized 13 moments (R13) methods could predict anti-Fourier heat transfer. The R13 moment method is described by a macroscopic set of transport equations of third order in the Knudsen number in a Chapman-Enskog approximation sense. The method combines the benefits of Grad-type moment equations and Burnett-type equations while omitting their deficiencies^7^. They showed that inverted heat flux could be related to the shear stress gradients in the domain, especially near the walls. Christou and Dadzie^4^ studied the micro-cavity heat transfer problem using a new continuum model named Bi-Velocity model which offers a modification to the standard NSF equations for simulating rarefied gas flows. They showed that cold-to-hot heat transfer could be simulated by the NSF equations modified with a new molecular-level diffusive flux associated with the gas molecule concentration.

The counter-gradient heat flux in pressure-driven plane Poiseuille flow has also been observed by John *et al*.^5^ and Akhlaghi *et al*.^6^. It was observed that cold-to-hot heat transfer occurs at the terminal sections of the channel in the case of cooling walls. Meanwhile, at the similar wall-inflow temperature condition, the cold-to-hot heat transfer exists along the channel centerline at the sufficiently high rarefied condition. Balaj *et al*.^8^ investigated the effect of shear work due to the velocity slip on the non-equilibrium heat transfer in a pressure-driven gas flow through a microchannel under the specified wall heat flux condition. They analyzed the counter-gradient heat flow phenomenon appearing at the cooling conditions and showed that the viscous dissipation and shear work play significant roles in the heat flow pattern at the rarefied condition. Balaj *et al*.^9^ classified three different heat flow patterns, i.e., complete hot-to-cold, the entire anti-Fourier, and localized anti-Fourier heat transfer in the case of pressure-driven gas flow through the microchannel under constant wall heat flux condition. It is observed that the competition between the contributions of temperature gradient and pressure gradient (shear stress) as well as viscous slip heating strongly affect the heat flow pattern in micro and nanochannels.

Recently, Roohi *et al*.^10^ detected the cold-to-hot heat transfer phenomenon in nano-scale isosceles triangular cavities. They showed that the anti-Fourier heat transfer is due to the competition between the Fourier term and shear stress gradient component of the heat flux constitutive relation at non-equilibrium regimes. A cold-to-hot heat transfer was observed near the moving lid at the slip regime, which was attributed to sharp bends in the velocity profile at the corners of the cavity, amplifying the velocity gradient contribution in the heat flux constitutive law (second order in Knudsen number) compared to the first order Fourier term^2,9^. With the further increase of the Knudsen number in the transition regime, they showed that the regions of cold-to-hot transfer almost occupy the whole domain while most of the heat lines are directed toward the inclined surface of the triangle.

In the current work, we investigate the cold-to-hot heat transfer in a lid-driven rarefied cavity flow based on the recently proposed decomposition method^11^ for the first time. The ballistic and collisional parts of the DSMC solution in a cavity are computed separately, and the contribution of each part to the overall heat transfer pattern is analyzed. A similar approach was used previously for the explanation of self-diffusion in long capillary tubes^12^ and rarefied gas short tube propulsion efficiency^13^. These decomposed forms (ballistic and collision parts) for the macroscopic flow properties were presented in^11,14,15^. Considering ballistic and collision parts of the heat flux and temperature by using a new indicator of cold-to-hot heat transfer reveals new understandings of the behavior of cold-to-hot heat transfer in rarefied gas flows in the cavity geometry.

## Methods

### DSMC solver

Direct Simulation Monte Carlo (DSMC) proposed by Bird is a numerical tool to solve the Boltzmann or Kac master equation based on the direct statistical simulation of the molecular processes described by the kinetic theory^16–19^. In the DSMC algorithm, a finite set of simulator particles is utilized to present a substantial number of real gas molecules. DSMC splits the ballistic movement and collision of simulated particles in each time step into two continuous processes. In this regard, simulation time step must be considered as a fraction of the mean collision time. Here, an improved version of DSMC2D.for^16^ was utilized. The variable hard sphere (VHS) model is applied as the collision model in all simulations. The cavity walls are treated as diffuse reflectors using full thermal accommodation coefficient, where the emission of the impinging molecules is not correlated with the pre-impingement state of the molecules. The outgoing velocity is randomly assigned according to a Maxwellian distribution determined by the wall temperature^16^. The outgoing velocity component normal to the surface is obtained from:

1 $${u}_{n}={V}_{mp}{[-\mathrm{log}({R}_{n})]}^{1/2}$$

The two tangential outgoing velocity components are calculated as:

2 $$\begin{array}{c}{u}_{{t}_{1}}={V}_{mp}\,\sin (2\pi R^{\prime} ){[-\mathrm{log}({R}_{t})]}^{1/2}\,\\ {u}_{{t}_{2}}={V}_{mp}\,\cos (2\pi R^{\prime} ){[-\mathrm{log}({R}_{t})]}^{1/2}\,\end{array}$$

where, *R*_*n*_, *R*_*t*_ and *R*′ are random numbers between 0 and 1. ${V}_{mp}$ is the most probable speed, e.g.:

3 $${V}_{mp}={[\frac{2k{T}_{w}}{m}]}^{1/2}$$

where *k* is the Boltzmann constant and *m* is molecular mass. In the case of lid moving at a velocity *U*_*w*_, the x-component of outgoing velocity is determined as:

4 $${u}_{t}={V}_{mpf}\,\sin (2\pi R^{\prime} ){[-\mathrm{log}({R}_{t})]}^{1/2}+{U}_{w}$$

Cell dimensions are chosen to be much smaller than gas mean free path^20^. Each cell is subsequently divided into two static subcells in each direction. Knudsen number is defined as the ratio of the mean free path (*λ*) to the cavity length scale (*L*), ${\rm{Kn}}=\lambda /L$^16^. The time step is taken to be a certain fraction of the mean collision time. Numbers of simulated particles are chosen such that there would be at least 20 particles in each cell. The sampling of the results starts as soon as the flow reaches steady state conditions. Argon gas, with a mass of *m* = 6.63 × 10^−26^ kg, molecular diameter of *d* = 4.17 × 10^−10^ m, viscosity-temperature index of *ω* = 0.74 at a reference temperature of *T*_*ref*_ = 273 K is employed as the working fluid for all simulations. Sample size, gathered after flow reached steady state condition and used to compute fluid properties, was at least 2 × 10^6^ per each cell.

### Ballistic and collision flow parts

The standard approach for analysis of the non-equilibrium state in a rarefied gas flow is built on revealing zones with slip, jump and creep of the macroscopic quantities (moments of the velocity distributed function) nearby solid (liquid) boundaries or asymmetries in shapes of the components of macroscopic variables such as velocity, temperature (shock waves, etc.). By an in-depth examination of the velocity distribution function, it can be found that these effects appear due to the existence of discontinuities and asymmetries in the form of distribution function in velocity space. The reasons for these effects in the vicinity of a space point might be explained on a molecular level by analysis of the particle motion. The particles arriving at this point from a given domain or boundary without collisions with other molecules carry information about corresponding zone while particles experiencing collisions with other particles undergo collision relaxation and exchange of energy. The decomposition of the distribution function into ballistic and collision parts allows giving a deeper insight into the non-equilibrium state existing in some zones of a rarefied gas flow in the entire range of Knudsen numbers. It can also facilitate to analyze separately and compare effects of molecular fluxes coming to every point in the flow from zones with different properties (ballistic part) and fluxes of particles undergoing a relaxation process due to collisions with other molecules (collision part). Thus, a kinetic solution at a specified point in the domain has two parts; 1) ballistic part which is produced by collisionless particles coming from the boundaries and 2) collision part which is resulted from sampling over particles after the occurrence of at least one collision after entering to the domain^11,14^. It is worth noting that both ballistic and collision parts are coupled. When a particle reflects from a boundary, it belongs to the ballistic part, and when it experiences at least one collision, it will be considered in the collisional part. At a point $(x,y)$, the dimensionless distribution function $g=g(x,y,\zeta )$ at a local point is defined as

5 $$g(x,y,\zeta )=\frac{1}{{N}_{s}V}\sum _{j=1}^{{N}_{s}}\sum _{i=1}^{{N}_{p}(j)}\delta (\zeta -{\zeta }_{i}(j))$$

where ${N}_{s}$ is the number of sampling, ${N}_{p}(j)$ is the number of particles in the cell at *j*-th sample, $V$ is the cell volume and *δ* is the Dirac delta function. $\zeta =[{\zeta }_{x},{\zeta }_{y},{\zeta }_{z}]$ denotes the molecular velocity vector. The decomposed form of *g* consists of ballistic (${g}^{(b)}$) and collision (${g}^{(c)}$) parts as bellow

6 $$g(x,y,\zeta )={g}^{(b)}(x,y,\zeta )+{g}^{(c)}(x,y,\zeta )$$

The prescribed decomposition of the particle distribution in a given cell of the computational grid with a center point $(x,y)$ can be implemented in the original DSMC algorithm as described in^11,14,15^. For example, a decomposed form of velocity components is given by:

7 $$\begin{array}{c}u(x,y)=\frac{\sum _{i=1}^{{{N}_{T}}^{(b)}}{\zeta }_{x,i}+\sum _{j=1}^{{{N}_{T}}^{(c)}}{\zeta }_{x,j}}{{N}_{T}}={u}^{(b)}(x,y)+{u}^{(c)}(x,y)\\ v(x,y)=\frac{\sum _{i=1}^{{{N}_{T}}^{(b)}}{\zeta }_{y,i}+\sum _{j=1}^{{{N}_{T}}^{(c)}}{\zeta }_{y,j}}{{N}_{T}}={v}^{(b)}(x,y)+{v}^{(c)}(x,y)\end{array}$$

where ${N}_{T}={{N}_{T}}^{(b)}+{{N}_{T}}^{(c)}$ is the total number of sampled particles. Ballistic and collision contributions to the number of sampled particles are denoted by ${{N}_{T}}^{(b)}$ and ${{N}_{T}}^{(c)}$, respectively.

In the current work, the sampling relations for ballistic and collision parts of temperature and heat flow is derived again in a slightly different form suitable for the following considerations. For a monatomic gas in a two dimensional flow, the macroscopic temperature in the cell is obtained by sampling over the kinetic energy of crossing particles^16,21^

8 $$T=\frac{1}{3k}[\frac{\sum _{i=1}^{{N}_{T}}{m}_{i}{{\zeta }_{i}}^{2}}{{N}_{T}}-\frac{\sum _{i=1}^{{N}_{T}}{m}_{i}}{{N}_{T}}({u}^{2}+{v}^{2})]$$

According to the definition of ballistic and collision decomposition for temperature, i.e., $T={T}^{(b)}+{T}^{(c)}$ and using Eq. (8), we have:

9 $$\begin{array}{c}{T}^{(b)}=\frac{1}{3k}[\frac{\sum _{j=1}^{{{N}_{T}}^{(b)}}{m}_{j}{{\zeta }_{j}}^{2}}{{N}_{T}}-\frac{\sum _{j=1}^{{{N}_{T}}^{(b)}}{m}_{j}}{{N}_{T}}({u}^{2}+{v}^{2})]\\ {T}^{(c)}=\frac{1}{3k}[\frac{\sum _{k=1}^{{{N}_{T}}^{(c)}}{m}_{k}{{\zeta }_{k}}^{2}}{{N}_{T}}-\frac{\sum _{k=1}^{{{N}_{T}}^{(c)}}{m}_{k}}{{N}_{T}}({u}^{2}+{v}^{2})]\end{array}$$

Heat flow components in the domain are given by:

10 $$\begin{array}{c}{q}_{x}=n(\frac{1}{2}\frac{\sum _{i=1}^{{N}_{T}}{m}_{i}{{\zeta }_{i}}^{2}{\zeta }_{x,i}}{{N}_{T}}-\frac{u}{2}\frac{\sum _{i=1}^{{N}_{T}}{m}_{i}{{\zeta }_{i}}^{2}}{{N}_{T}}-u\frac{\sum _{i=1}^{{N}_{T}}{m}_{i}{{\zeta }_{x,i}}^{2}}{{N}_{T}}+{u}^{3}\frac{\sum _{i=1}^{{N}_{T}}{m}_{i}}{{N}_{T}}-v\frac{\sum _{i=1}^{{N}_{T}}{m}_{i}{\zeta }_{x,i}{\zeta }_{y,i}}{{N}_{T}}+u{v}^{2}\frac{\sum _{i=1}^{{N}_{T}}{m}_{i}}{{N}_{T}})\\ {q}_{y}=n(\frac{1}{2}\frac{\sum _{i=1}^{{N}_{T}}{m}_{i}{{\zeta }_{i}}^{2}{\zeta }_{y,i}}{{N}_{T}}-\frac{v}{2}\frac{\sum _{i=1}^{{N}_{T}}{m}_{i}{{\zeta }_{i}}^{2}}{{N}_{T}}-u\frac{\sum _{i=1}^{{N}_{T}}{m}_{i}{\zeta }_{x,i}{\zeta }_{y,i}}{{N}_{T}}+{u}^{2}v\frac{\sum _{i=1}^{{N}_{T}}{m}_{i}}{{N}_{T}}-v\frac{\sum _{i=1}^{{N}_{T}}{m}_{i}{{\zeta }_{y,i}}^{2}}{{N}_{T}}+{v}^{3}\frac{\sum _{i=1}^{{N}_{T}}{m}_{i}}{{N}_{T}})\end{array}$$

where *n* is the cell local number density. The decomposed form of heat flow components is defined as

11 $$\begin{array}{c}{q}_{x}={{q}_{x}}^{(b)}+{{q}_{x}}^{(c)}\\ {q}_{y}={{q}_{y}}^{(b)}+{{q}_{y}}^{(c)}\end{array}$$

From Eqs (10 and 11), they read

12 $$\begin{array}{c}{{q}_{x}}^{(b)}=n(\frac{1}{2}\frac{\sum _{j=1}^{{{N}_{T}}^{(b)}}{m}_{j}{{\zeta }_{j}}^{2}{\zeta }_{x,j}}{{N}_{T}}-\frac{u}{2}\frac{\sum _{j=1}^{{{N}_{T}}^{(b)}}{m}_{j}{{\zeta }_{j}}^{2}}{{N}_{T}}-u\frac{\sum _{j=1}^{{{N}_{T}}^{(b)}}{m}_{j}{{\zeta }_{x,j}}^{2}}{{N}_{T}}+{u}^{3}\frac{\sum _{j=1}^{{{N}_{T}}^{(b)}}{m}_{j}}{{N}_{T}}-v\frac{\sum _{j=1}^{{{N}_{T}}^{(b)}}{m}_{j}{\zeta }_{x,j}{\zeta }_{y,j}}{{N}_{T}}+u{v}^{2}\frac{\sum _{j=1}^{{{N}_{T}}^{(b)}}{m}_{j}}{{N}_{T}})\\ {{q}_{x}}^{(c)}=n(\frac{1}{2}\frac{\sum _{k=1}^{{{N}_{T}}^{(c)}}{m}_{k}{{\zeta }_{k}}^{2}{\zeta }_{x,k}}{{N}_{T}}-\frac{u}{2}\frac{\sum _{k=1}^{{{N}_{T}}^{(c)}}{m}_{k}{{\zeta }_{k}}^{2}}{{N}_{T}}-u\frac{\sum _{k=1}^{{{N}_{T}}^{(c)}}{m}_{k}{{\zeta }_{x,k}}^{2}}{{N}_{T}}+{u}^{3}\frac{\sum _{k=1}^{{{N}_{T}}^{(c)}}{m}_{k}}{{N}_{T}}-v\frac{\sum _{k=1}^{{{N}_{T}}^{(c)}}{m}_{k}{\zeta }_{x,k}{\zeta }_{y,k}}{{N}_{T}}+u{v}^{2}\frac{\sum _{k=1}^{{{N}_{T}}^{(c)}}{m}_{k}}{{N}_{T}})\\ {{q}_{y}}^{(b)}=n(\frac{1}{2}\frac{\sum _{j=1}^{{{N}_{T}}^{(b)}}{m}_{j}{{\zeta }_{j}}^{2}{\zeta }_{y,j}}{{N}_{T}}-\frac{v}{2}\frac{\sum _{j=1}^{{{N}_{T}}^{(b)}}{m}_{j}{{\zeta }_{j}}^{2}}{{N}_{T}}-u\frac{\sum _{j=1}^{{{N}_{T}}^{(b)}}{m}_{j}{\zeta }_{x,j}{\zeta }_{y,j}}{{N}_{T}}+{u}^{2}v\frac{\sum _{j=1}^{{{N}_{T}}^{(b)}}{m}_{j}}{{N}_{T}}-v\frac{\sum _{j=1}^{{{N}_{T}}^{(b)}}{m}_{j}{{\zeta }_{y,j}}^{2}}{{N}_{T}}+{v}^{3}\frac{\sum _{j=1}^{{{N}_{T}}^{(b)}}{m}_{j}}{{N}_{T}})\\ {{q}_{y}}^{(c)}=n(\frac{1}{2}\frac{\sum _{k=1}^{{{N}_{T}}^{(c)}}{m}_{k}{{\zeta }_{k}}^{2}{\zeta }_{y,k}}{{N}_{T}}-\frac{v}{2}\frac{\sum _{k=1}^{{{N}_{T}}^{(c)}}{m}_{k}{{\zeta }_{k}}^{2}}{{N}_{T}}-u\frac{\sum _{k=1}^{{{N}_{T}}^{(c)}}{m}_{k}{\zeta }_{x,k}{\zeta }_{y,k}}{{N}_{T}}+{u}^{2}v\frac{\sum _{k=1}^{{{N}_{T}}^{(c)}}{m}_{k}}{{N}_{T}}-v\frac{\sum _{k=1}^{{{N}_{T}}^{(c)}}{m}_{k}{{\zeta }_{y,k}}^{2}}{{N}_{T}}+{v}^{3}\frac{\sum _{k=1}^{{{N}_{T}}^{(c)}}{m}_{k}}{{N}_{T}})\end{array}$$

Using the decompositions mentioned above, the well-known cold-to-hot heat transfer problem in non-equilibrium rarefied condition is investigated from ballistic and collision viewpoints for temperature and heat flux fields in the cavity flow.

## Results and Discussion

**Figure 1:**
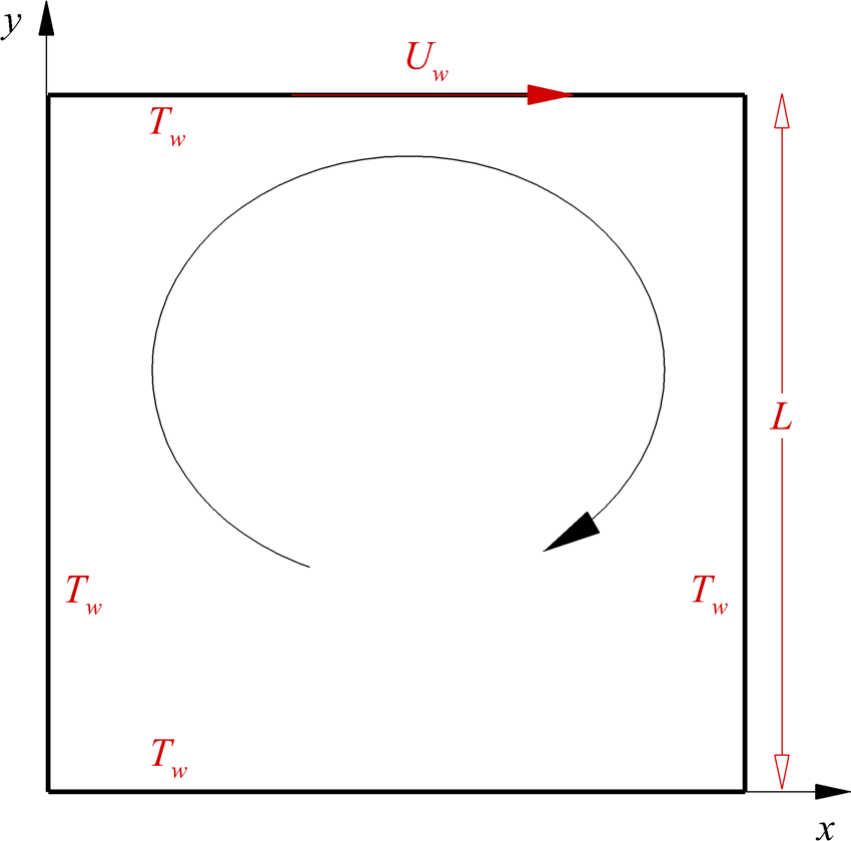
Lid-driven cavity with a length of *L*, wall temperature of *T*_*w*_ and lid velocity of *U*_*w*_.

**Figure 2:**
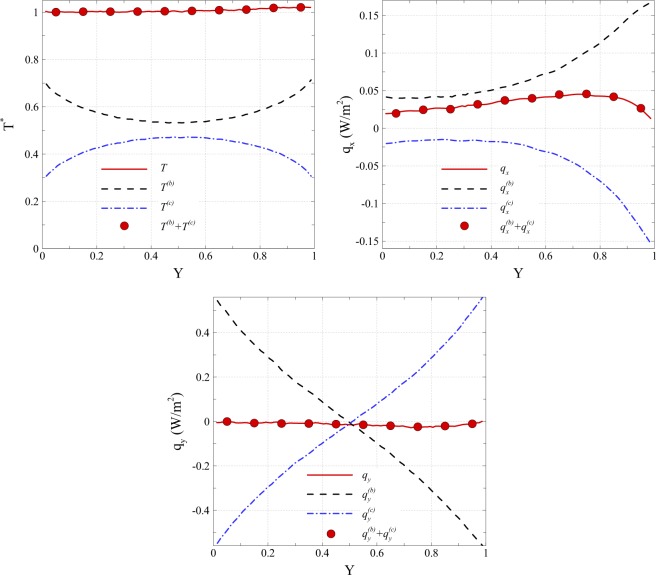
Ballistic, collision, and overall profiles of temperature and heat flux components over the line *x/L* = 0.5 in case ${\rm{Kn}}=1$.

The lid-driven cavity considered in this study is shown in Fig. 1. The lid moves in the positive x-direction at ${U}_{w}=100\,m/s$. Rarefied gas flow in the cavity in slip and transitional flow regimes, i.e., for a range of Knudsen numbers Kn = 0.005–1, is considered. The temperature of the walls is set at ${T}_{w}=300K$. Figure 2 presents the accuracy of proposed ballistic and collision decompositions for temperature and heat flow flux components. The profiles have been obtained for the case Kn = 1 on the vertical line in the middle of the cavity, i.e. $x/L=0.5$. The circle symbols correspond to values of a superposition of ballistic and collision parts (Eqs 9 and 12). As can be seen, the summations of ballistic and collisional contributions are in excellent agreement with overall values obtained using Eqs (8 and 10). This proofs that the imposed decomposition in our DSMC solver works accurately. However, the decomposition error may increase if the decomposed parameter is extremely low. For example, in the case of heat flux decomposition, the error may increase due to smaller values of heat flux at larger Kn numbers. In all the test cases of this paper, decomposition error is estimated to be less than 5%.

**Figure 3:**
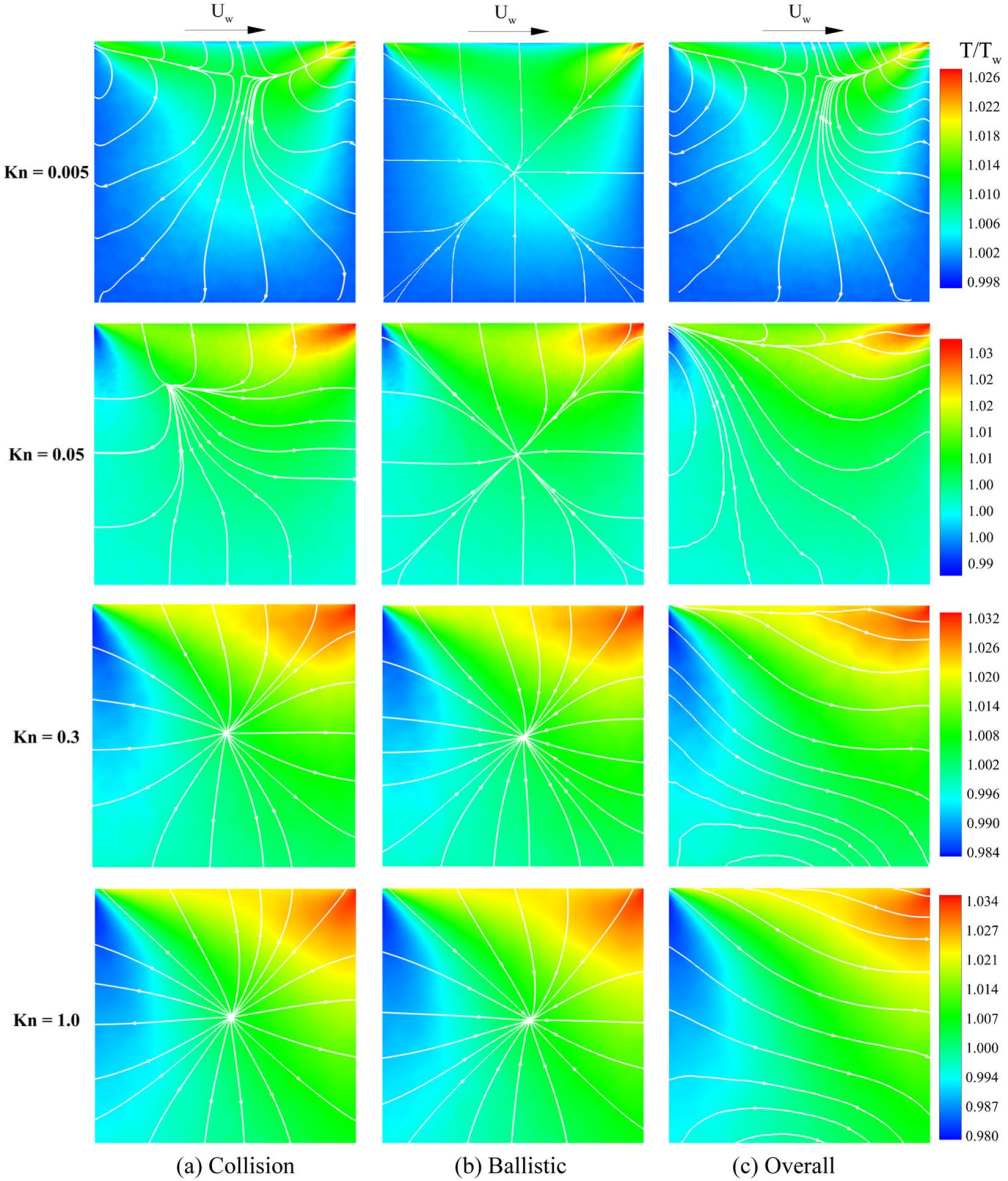
Ballistic, collision, and overall heat flow lines overlaid on the overall temperature field for different Knudsen numbers.

Figure 3 shows the heat flow lines (Eqs 10 and 12) overlaid on the overall temperature contours from Eq. (8). Heat flow lines are plotted for overall (right), ballistic (middle), and collision (left) contributions and for a broad range of Knudsen numbers from slip (Kn = 0.005) to mid transitional flow (Kn = 1). According to the results, both collision and ballistic parts contribute to overall heat transfer. Collision part is dominant at low Knudsen numbers, and it determines the pattern of overall heat flow as it is seen for the Kn = 0.005 case. Ballistic part starts influencing the overall heat flow pattern as rarefaction effects become stronger. According to the figure, there are cold-to-hot heat transfer regions for cases with Kn > 0.005. The cold-to-hot heat transfer phenomenon magnifies for larger rarefaction in the gas flow. The heat lines of the collisional part demonstrate that a source line with an orientation towards boundaries is originated. The source is located in the region near the moving lid at the slip condition, i.e., Kn = 0.005. With increasing the Knudsen number, this line transforms into a source point and moves to the central region of the cavity.

Contrary to the collisional part, there is a sink point in the ballistic heat flow pattern approximately close to the geometrical center of the cavity that absorbs heat lines from boundaries. In fact, ballistic particles influence the domain after reflection from solid surfaces. In other words, the direction of energy transfer by ballistic particles is from boundaries to the middle areas of the domain. Therefore, there is a sink point in the ballistic heat transfer pattern in the middle. On the other hand, the concentration of non-ballistic flow is higher in the middle regions of the domain. Hence, the energy transfer by non-ballistic particles is from the central region to the boundaries. This leads to the emerging of a source line or source point in the collisional heat flow pattern.

The competition between the heat source from collisional part and heat sink from ballistic part makes the overall heat flow structure. Considering overall temperature variations, both collision and ballistic parts exhibit hot-to-cold and cold-to-hot heat transfer in their heat flow patterns. Based on the results shown in Fig. 3, both ballistic and collision heat parts contribute to cold-to-hot heat transfer mechanism. Collisional part leads to cold-to-hot heat transfer on the right side, and ballistic part leads to anti-Fourier heat transfer on the left side.

**Figure 4:**
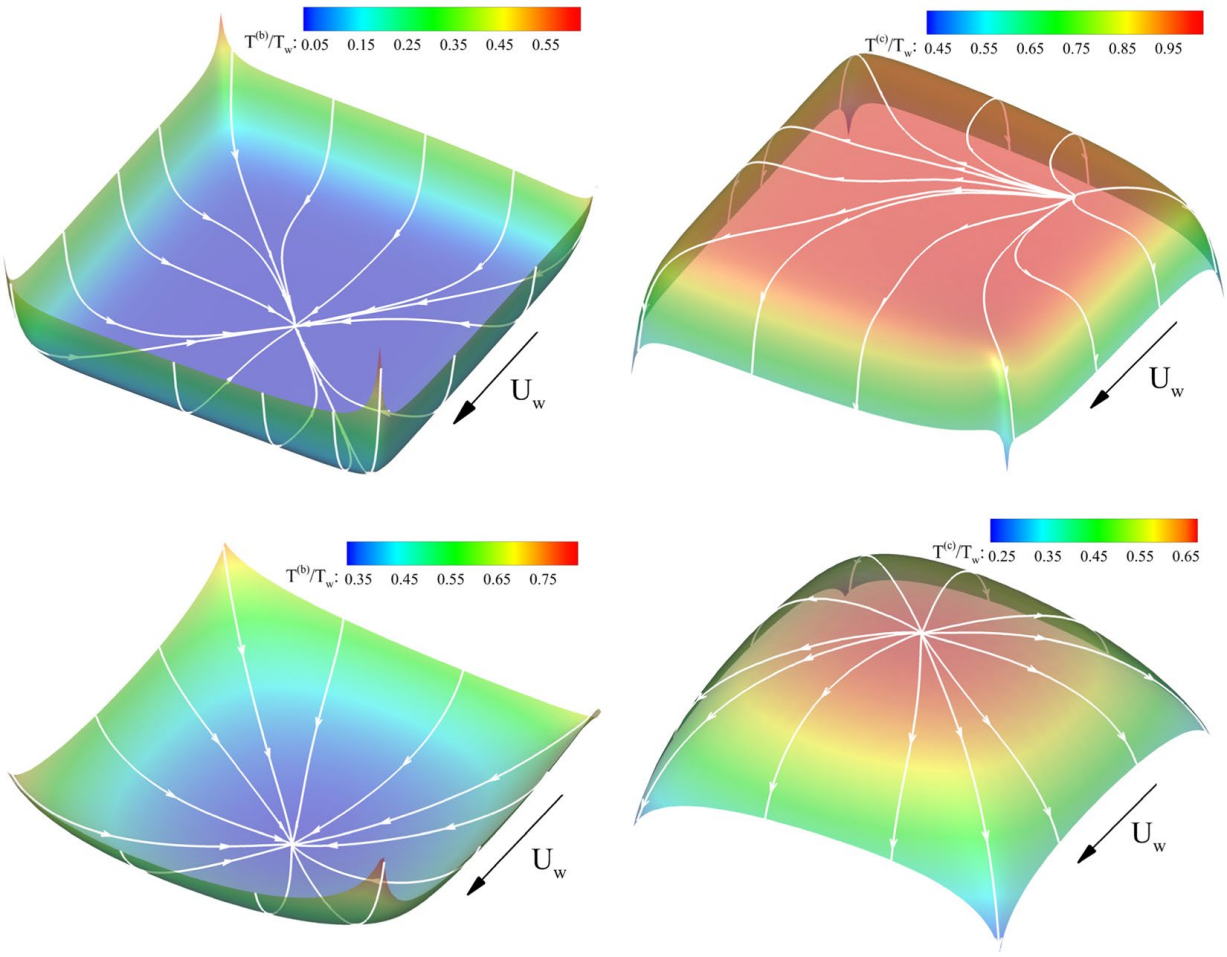
Ballistic heat flow lines overlaid on the ballistic temperature field (left) and collision heat flow lines overlaid on the collision temperature field (right) for ${\rm{Kn}}=0.05$ (up) and ${\rm{Kn}}=0.5$ (down).

Figure 4 investigates the cold-to-hot phenomena for collision and ballistic flow parts separately, i.e., it considers collision heat flow pattern concerning the collision temperature field and ballistic heat flow pattern with respect to the ballistic temperature field. Hence, ballistic heat flow lines overlaid on the ballistic temperature field (left) and collision heat flow lines overlaid on the collision temperature fields (right) are presented in the figure at two different levels of flow rarefactions, i.e., Kn = 0.05 (up) and Kn = 0.5 (down). Remarkably, it can be inferred that when considered separately, the ballistic and collision parts of heat flux are directed from hot-to-cold in each point of the corresponding ballistic and collision temperature fields. Consequently, considering separately, there are no zones with cold-to-hot heat transfer from this viewpoint. The temperature contours reveal that ballistic temperature has a greater contribution to the temperature field near the boundaries and drops as the heat lines approach the cavity center. Contrary to the ballistic part, maximum and minimum values of the collisional temperature are located at the cavity center and near the boundaries, respectively. As flow approaches to the middle area of the domain from the boundaries, the ballistic temperature decreases and collisional counterpart increases. This indicates an adverse direction for the temperature gradient of ballistic and collisional parts.

Further numerical analysis showed that this observation could be generalized to all degrees of flow rarefaction. Accordingly, one can conclude that ballistic (collision) heat transfer occurs in opposite direction of ballistic (collision) temperature gradient at all degrees of flow rarefaction. When the above considerations are put together with the overall solution of the heat flux, it becomes clear that the cold-to-hot transfer in a rarefied cavity flow is a result of a subtle interplay between ballistic and collisional parts of the decomposed solution. This general rule can be used to define a new indicator of cold-to-hot transfer.

We define *κ* as an anti-Fourier heat transfer criterion to determine the cold-to-hot heat transfer regions with a higher resolution. It is defined as the dot product of the normalized heat flow vector and normalized temperature gradient vector and could be applied it to the overall, ballistic and collision parts of the corresponding flow fields. For the overall flow field it reads:

13 $$\kappa =\frac{{\bf{q}}}{{({\bf{q}}\cdot {\bf{q}})}^{1/2}}\cdot \frac{\overrightarrow{\nabla T}}{{(\overrightarrow{\nabla T}\cdot \overrightarrow{\nabla T})}^{1/2}}$$

where ${\bf{q}}=({q}_{x},{q}_{y})$ is heat flow vector and $\overrightarrow{\nabla T}$ is temperature gradient vector. According to Eq. (13), *κ* is a real number between −1 and 1. Positive values *κ* indicate that heat flow vector has a component in the direction of temperature gradient vector and vice versa. Based on Eq. (13), *κ* = 1 indicates pure cold-to-hot and *κ* = −1 represents pure hot-to-cold heat transfer. Also, $0 < \kappa  < 1$ and $-1 < \kappa  < 0$ correspond to partial cold-to-hot and partial hot-to-cold heat transfer, respectively.

**Figure 5:**
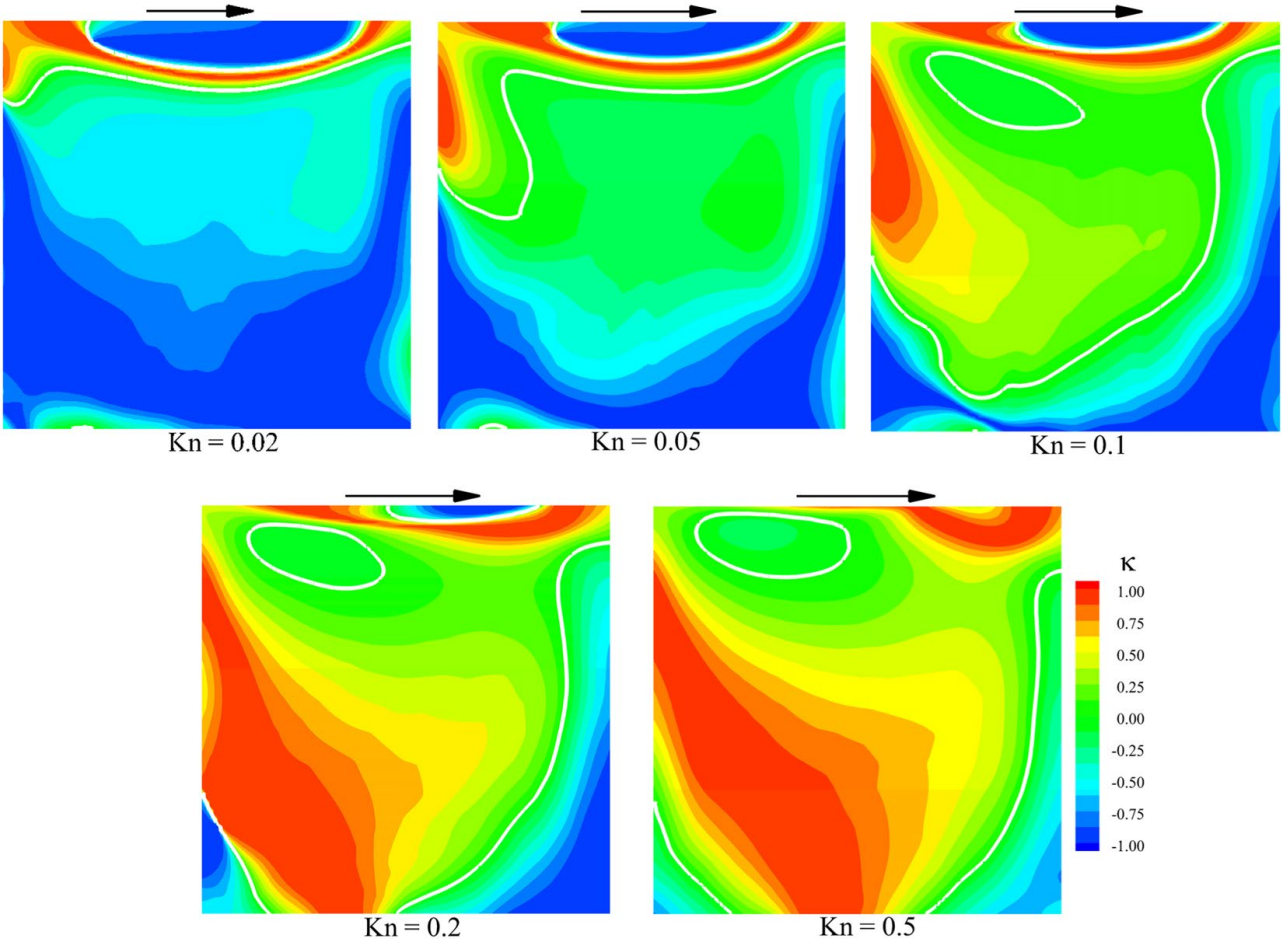
Contours of the parameter *κ* based on overall temperature and overall heat flow.

Figure 5 shows the contours of the *κ* parameter at different flow rarefactions. The boundary between cold-to-hot and hot-to-cold heat transfer regions is illustrated by white lines to demonstrate the distinction between the regions. In the low rarefaction cases, the regions of cold-to-hot transfer are negligible and limited to the upper side and upper left corner of the cavity. As Knudsen number increases, the upper side region moves left near the upper right corner. Also, cold-to-hot transfer region expands significantly from the upper left corner to the central region of the cavity. Figure 5 indicates that the region of cold-to-hot heat transfer is symmetric about $x/L=0.5$ at low rarefaction condition and it expands as Knudsen increases. According to Fig. 3, the pattern of overall heat flow is approximately symmetric with respect to the $x/L=0.5$ at Kn = 0.02. As Knudsen number increases, the heat flow lines align with the direction of lid movement. At low rarefied condition, diffusion from the intermolecular collision is dominant compared to the gas-surface interaction; hence, the effect of lid movement is negligible in the pattern of heat flux. With the increase in Knudsen number, the gas-surface molecular collision is in more effect compared to the intermolecular collision. Thus, the influence of lid movement in the overall heat flow lines pattern dominates as wall-reflected particles could move farther from the wall experiencing fewer intermolecular interactions.

According to the discussion mentioned above, cold-to-hot heat transfer in an isothermal cavity occurs in the presence of rarefaction effects. Aligned with lid movement, a temperature gradient for all level of flow rarefaction is observed. At the continuum limit, the pattern of the temperature gradient dictates the pattern of heat flow as Fourier law which leads to hot-to-cold heat transfer. However, in the presence of rarefaction effects, the contribution of the gas-surface interaction aligns the heat flow lines with the direction of lid movement which leads to cold-to-hot heat transfer.

**Figure 6:**
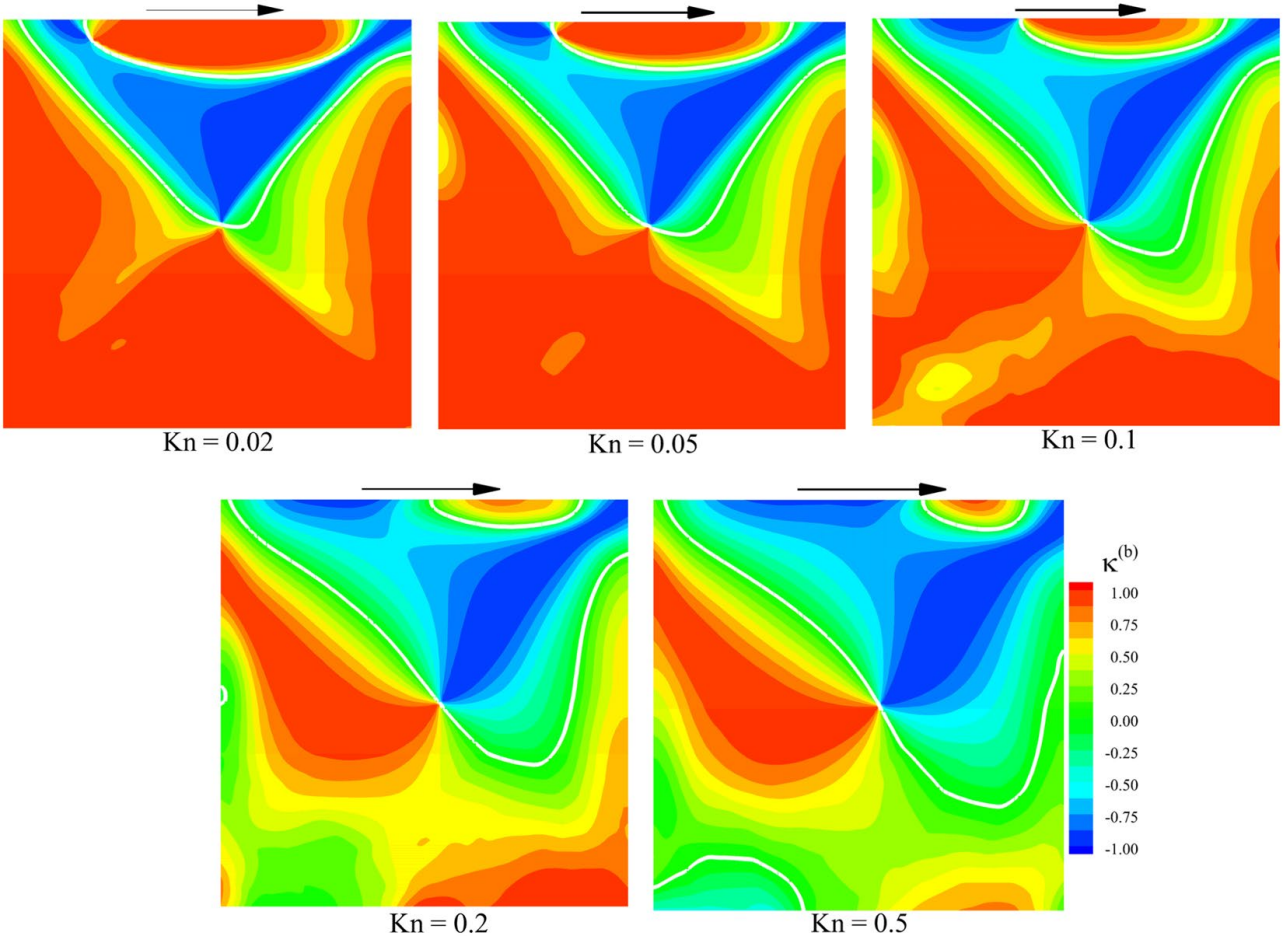
Contours of parameter κ^(b)^ fields based on overall temperature and ballistic heat flow.

Similar to Eq. (13), the ballistic ${\kappa }^{(b)}$ is defined as the dot product of the normalized ballistic heat flow vector and normalized overall temperature gradient vector:

14 $${\kappa }^{(b)}=\frac{{{\bf{q}}}^{(b)}}{{({{\bf{q}}}^{(b)}\cdot {{\bf{q}}}^{(b)})}^{1/2}}\cdot \frac{\overrightarrow{\nabla T}}{{(\overrightarrow{\nabla T}\cdot \overrightarrow{\nabla T})}^{1/2}}$$

where ${{\bf{q}}}^{(b)}=({{q}_{x}}^{(b)},{{q}_{y}}^{(b)})$ is ballistic heat flow vector. According to Eq. (14), at locations with ${\kappa }^{(b)} > 0$, ballistic part of motion magnifies cold-to-hot heat transfer and where ${\kappa }^{(b)} < 0$ ballistic part of motion weakens cold-to-hot heat transfer. Figure 6 depicts the contours of ${\kappa }^{(b)}$ at different rarefied conditions. The regions of cold-to-hot and hot-to-cold heat transfer are distinguished by white lines. The ${\kappa }^{(b)}$ contours are symmetric at Kn = 0.02, and the effect of lid movement direction is negligible. As Knudsen number increases the symmetry is disrupted by lid movement due to the considerable effect of gas-surface interactions at rarefied conditions. At low rarefaction cases, i.e. ${\rm{Kn}}\le 0.05$, there are extended regions of cold-to-hot heat transfer close to the top, right, and left sides. However, it should be mentioned that the magnitude of heat transfer in these cases is minimal and their contribution to the overall anti-Fourier heat transfer is negligible. It is seen that the cold-to-hot region in the top side approaches the upper right corner as Kn increases. Correspondingly, hot-to-cold heat transfer region is shifted towards left as the flow becomes more rarefied. It should be reminded that cold-to-hot heat transfer regions are near the walls at lower rarefaction conditions. At these conditions, the temperature rises away from the wall, but the direction of ballistic particles is also away from the wall. As rarefaction increases, the effect of lid motion overcomes intermolecular collisions. The rightward lid movement causes the gas density and intermolecular collision frequency to decrease near the left corner and increase adjacent to the right corner. This leads to an extension of cold-to-hot heat transfer region in the left side due to the accumulation of ballistic molecules and a shrinkage in the right side due to the accumulation of collisional particles.

**Figure 7:**
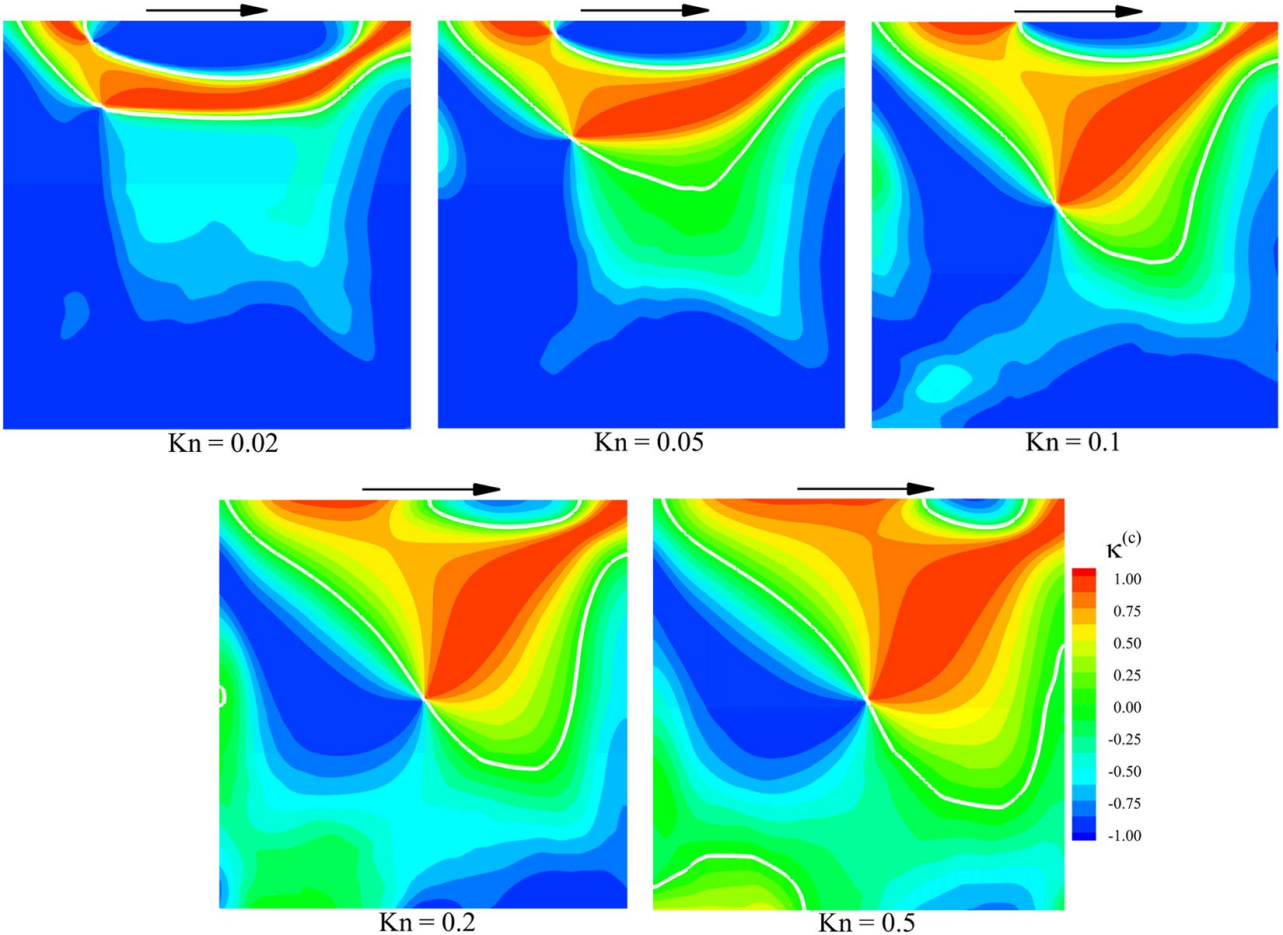
Contours of parameter κ^(c)^ based on the overall temperature and collision heat flow.

Similarly, the parameter ${\kappa }^{(c)}$ is defined as the dot product of normalized collision heat flow vector and normalized overall temperature gradient vector:

15 $${\kappa }^{(c)}=\frac{{{\bf{q}}}^{(c)}}{{({{\bf{q}}}^{(c)}\cdot {{\bf{q}}}^{(c)})}^{1/2}}\cdot \frac{\overrightarrow{\nabla T}}{{(\overrightarrow{\nabla T}\cdot \overrightarrow{\nabla T})}^{1/2}}$$

where ${{\bf{q}}}^{(c)}=({{q}_{x}}^{(c)},{{q}_{y}}^{(c)})$ is collision heat flow vector. For ${\kappa }^{(c)} > 0$, collision part of heat transfer represents cold-to-hot transfer and for ${\kappa }^{(c)} < 0$, it depicts hot-to-cold. Figure 7 depicts the contours of ${\kappa }^{(c)}$ at different rarefied degrees. As before, the regions of cold-to-hot and hot-to-cold heat transfer are distinguished by white lines. There exists a narrow and approximately symmetric anti-Fourier region around $x/L=0.5$ near the top of the cavity for ${\rm{Kn}}\le 0.02$. This symmetrical behavior is similar to those observed in Figs 5 and 6. At higher rarefaction conditions, the pattern of cold-to-hot and hot-to-cold heat transfer regions changes due to the contribution of ballistic particles coming from the lid. As the Knudsen number increases, this region extends downward and occupies the right side of the cavity. Comparison of Figs 6 and 7 indicates that for all degrees of rarefaction, approximately everywhere heat transfer due to the ballistic flow is in cold-to-hot mode while its collisional counterpart shows a hot-to-cold pattern. It is due to the fact that the magnitude of overall heat flux in the isothermal cavity is small. Therefore, the vectors of heat flux for ballistic and collisional flow are approximately of the same magnitude, but in different directions. This fact can also be observed in Fig. 3. Moreover, as observed in Fig. 5, for Knudsen numbers higher than ${\rm{Kn}} > 0.1$, the anti-Fourier heat transfer region occupies the whole bulk region of the domain, unlike the low Knudsen number cases in which this area is only limited to the near wall regions. According to the Fig. 7, it can be concluded that the central extension of anti-Fourier heat transfer region to the bulk domain is due to the ballistic part of the heat transfer.

**Figure 8:**
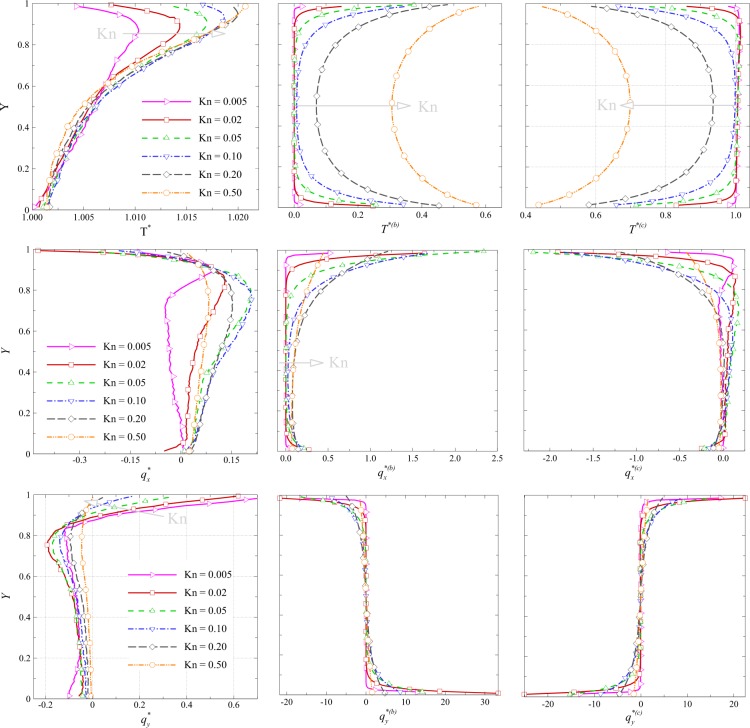
Vertical profiles for overall/ballistic/collisional values of temperature and heat flux components at *x*/*L* = 0.5 at various Knudsen numbers.

**Figure 9:**
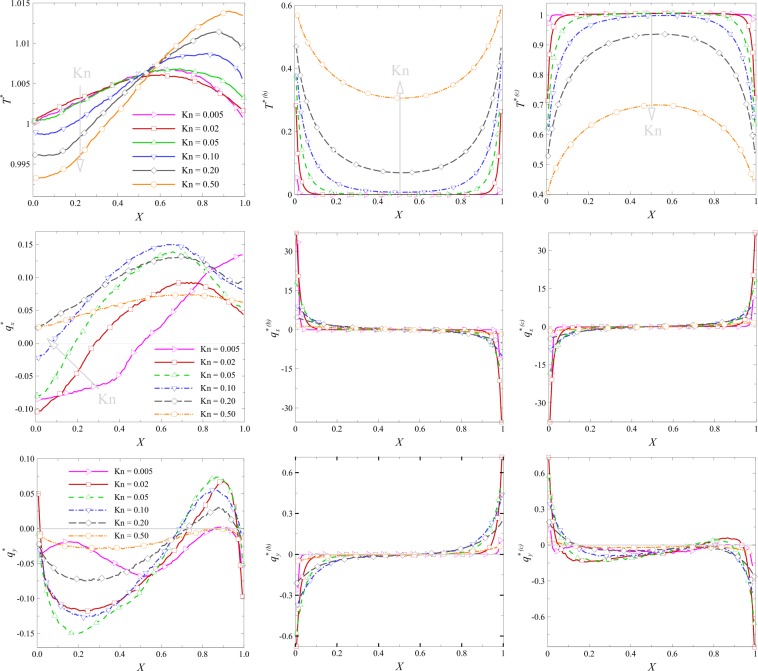
Horizontal profiles for overall/ballistic/collision values of temperature and heat flux components at *y*/*L* = 0.5 at various Knudsen numbers.

The vertical and horizontal profiles of overall/ballistic/collision heat flux components and temperature are reported in Figs 8 and 9 for different values of Knudsen number. Vertical profiles at $x/L=0.5$ are plotted in Fig. 8. Temperature profiles indicate that the values of ballistic temperature near the top and down sides are higher than those in middle of the domain. Close to the boundaries, the chance of collision for a ballistic particle increases and consequently the ballistic contributions decreases. However, with an increase in the Knudsen number, the intermolecular collision decreases and ballistic contributions increase. Ballistic and collision temperature profiles also indicate a type of boundary layer, especially at low rarefied flow condition, i.e. ${\rm{Kn}} < 0.1$.

The high-temperature zone is concentrated near the top side and moves up as ${\rm{Kn}}$ increases. This is because of the viscous heating effects near the wall which increases with the increase in the Knudsen number. Therefore, at higher Knudsen number where viscous heating effects are stronger, maximum temperature is observed near to the wall. Non-zero values of ballistic/collisional heat flux components occur near the boundaries. The sign of ballistic and collisional heat flux components are approximately opposite. This leads to an overall heat flux profile with sign variation. As flow rarefaction increases, the regions of maximum ${q}_{x}$ and ${q}_{y}$ move down and up, respectively. As rarefaction increases, the high-velocity region near the wall penetrates downward^22^. Accordingly, the maxima for x-component of the heat flux is shifted down. With the increase in Knudsen number and therefore decrease in velocity magnitude near the wall, diffusion in normal direction increases which makes regions with a higher normal component of the heat flux to be shifted upwards. Horizontal profiles of overall/ballistic/collision temperature and heat flux components at the location $y/L=0.5$ are reported in Fig. 9. Similar to Fig. 8, ballistic/collision temperature profile indicates the presence of a boundary layer at ${\rm{Kn}} < 0.1$ within which ballistic contribution on temperature is negligible. The signs of ballistic and collision heat flux components are opposite. The point with maximum ${q}_{x}$ moves left for ${\rm{Kn}} < 0.1$ and then right for ${\rm{Kn}} > 0.1$ with an increase in the Knudsen number. The profiles of ballistic/collision heat flux components are approximately asymmetric with respect to the centerline of the cavity. The aforementioned boundary layer for ballistic contribution could also be detected in profiles of heat flux components.

Considering Figs 8 and 9, the temperature and heat flux behavior seems to be different at smaller Knudsen numbers compared to higher rarefaction conditions. The reason may lay in different mechanism contributing to thermal and heat flux behavior at different rarefaction regimes. At very low Kn, temperature and heat flux are governed by the Fourier law in most part, while the non-equilibrium terms contributing in thermal and heat flux fields show up at moderate and higher Kn conditions. Sone^23^ showed that a weakly nonlinear form of the Boltzmann equation assigns the second derivative of velocity in the constitutive law of the heat flux in addition to the Fourier term. Mohammadzadeh *et al*.^2^ and Roohi *et al*.^10^ showed that the contribution of this term enhances in strength at higher Knudsen numbers in the cavity flow and considerably influences the pattern of heat flux, e.g., its contribution even changes the heat flux patterns from hot-to-cold to cold-to-hot. In parallel, the reason of change of behavior in heat flux field from small Kn to moderate Kn here could be greater contributions of non-equilibrium terms such as the second derivative of velocity. This is confirmed by the analysis of ballistic and collisional parts. In Figs 8 and 9, the plots of ballistic and collision parts clearly delineate the thickness of the Knudsen layer. At small Knudsen number ($Kn=0.005-0.01$), a thin Knudsen layer is observed close to the cavity walls. The ballistic part rapidly decays and goes to zero because of the enhanced binary collision rate. At the same time the, collisional part dominates and goes to one. At moderate Knudsen number Kn = 0.5, the thickness of the Knudsen layer is enhanced and both ballistic and collisional parts are distributed in the whole domain.

## Conclusions

For the first time, anti-Fourier heat transfer in a lid-driven cavity flow was considered using a ballistic/collision flow decomposition in the DSMC method under various rarefied flow conditions. The flow’s ballistic part makes a negligible contribution in the near continuum condition. Thus, the overall temperature and heat flux fields could be estimated with the collisional part. It was demonstrated that both the ballistic and collision parts of the flow contribute to the cold-to-hot heat transfer occurrence that takes place at different regions of the domain. The patterns of overall as well as collisional and ballistic heat transfer were approximately symmetric at nearly $x/L=0.5$ at extremely low rarefied conditions due to the dominance of intermolecular collisions. As the flow becomes further rarefied, the heat flow lines align with the direction of lid movement, leading to the cold-to-hot heat transfer. Ballistic particles enter the cavity’s central region after reflection from a solid surface, and this leads to the formation of a sink point in the ballistic heat transfer pattern. Moreover, the concentration of non-ballistic flow in the middle regions of the domain was found to be higher. Hence, the energy transferred by non-ballistic particles was directed from the middle region to the boundaries. This contributed to the appearance of a source line or source point in the collisional heat flow pattern. Based on the ballistic temperature field, the ballistic heat transfer can always be of Fourier type for all considered Knudsen numbers. This is also true for the collision part. Using an anti-Fourier heat transfer criterion, the regions of cold-to-hot heat transfer were investigated with higher resolution for overall, ballistic, and collision parts. As the flow became further rarefied, anti-Fourier heat transfer regions dominated by ballistic flow parts shrank while those affected by collision flow parts expanded. Vertical and horizontal profiles of overall/ballistic/collision temperature and heat flux components revealed a boundary layer, within which, the ballistic flow part was found to be negligible at low rarefied conditions.

## References

[1] John D, Gu XJ, Emerson DR (2010). Investigation of heat and mass transfer in a lid-driven cavity under nonequilibrium flow conditions. Numerical Heat Transfer Part B.

[2] Mohammadzadeh A, Roohi E, Niazmand H, Stefanov S, Myong RS (2012). Thermal and second-law analysis of a micro- or nanocavity using direct-simulation Monte Carlo. Physical Review E.

[3] Rana A, Torrilhon M, Struchtrup H (2013). A robust numerical method for the R13 equations of rarefied gas dynamics: Application to lid driven cavity. Journal of Computational Physics.

[4] Christou C, Dadzie SK (2017). An investigation of heat transfer in a cavity flow in the noncontinuum regime. Journal of Heat Transfer.

[5] John B, Gu XJ, Emerson DR (2013). Nonequilibrium gaseous heat transfer in pressure-driven plane Poiseuille flow. Physical Review E.

[6] Akhlaghi H, Roohi E, Balaj M, Dadzie SK (2014). Wall heat transfer effects on the hydro/thermal behaviour of Poiseuille flow in micro/nanochannels. Physics of Fluids.

[7] Struchtrup H, Torrilhon M (2008). Higher-order effects in rarefied channel flows. Physical review E.

[8] Mahdavi AM, Roohi E (2015). Investigation of cold-to-hot transfer and thermal separation zone through nano step geometries. Physics of Fluids.

[9] Balaj M, Roohi E, Mohammadzadeh A (2017). Regulation of anti-Fourier heat transfer for non-equilibrium gas flows through micro/nanochannels. International Journal of Thermal Sciences.

[10] Roohi E, Shahabi V, Bagherzadeh A (2018). On the vortical characteristics and cold-to-hot transfer of rarefied gas flow in a lid driven isosceles orthogonal triangular cavity with isothermal walls. International Journal of Thermal Sciences.

[11] Vargas M, Tatsios G, Valougeorgis D, Stefanov S (2014). Rarefied gas flow in a rectangular enclosure induced by non-isothermal walls. Physics of Fluids.

[12] Pollard WG, Present RD (1948). On gaseous self-diffusion in long capillary tubes. Physical Review.

[13] Lilly TC, Gimelshein SF, Ketsdever AD, Markelov GN (2006). Measurements and computations of mass flow and momentum flux through short tubes in rarefied gases. Physics of Fluids.

[14] Tatsios G, Stefanov SK, Valougeorgis D (2015). Predicting the Knudsen paradox in long capillaries by decomposing the flow into ballistic and collision parts. Physical Review E.

[15] Tatsios G, Vargas MH, Stefanov SK, Valugeorgis D (2016). Nonequilibrium gas flow and heat transfer in a heated square microcavity. Heat Transfer Engineering.

[16] Bird, G. A. in *Molecular gas dynamics and the direct simulation of gas flows* 1^st^ edition (ed, Bird, G. A.) (Clarendon Press, 1994).

[17] Roohi E, Stefanov S (2016). Collision partner selection schemes in DSMC: From micro/nano flows to hypersonic flows. Physics Reports.

[18] Akhlaghi H, Roohi E, Stefanov S (2018). On the Effects of Successively Repeated Collisions in the No Time Counter Collision Scheme in DSMC. Computers & Fluids.

[19] Roohi E, Stefanov S, Shoja-Sani A, Ejraei H (2018). A Generalized Form of the Bernoulli Trial Collision Scheme in DSMC: Derivation and Evaluation. Journal of Computational Physics.

[20] Hadjiconstantinou NG (2000). Analysis of discretization in the direct simulation Monte Carlo. Physics of Fluids.

[21] Bird, G. A. in *The DSMC Method* 1^st^ edition (ed, Bird, G. A.) (CreateSpace Independent Publishing Platform, 2013).

[22] Akhlaghi H, Roohi E, Stefanov S (2012). A new iterative wall heat flux specifying technique in DSMC for heating/cooling simulations of MEMS/NEMS. International Journal of Thermal Sciences.

[23] Sone, Y. in *Molecular Gas Dynamics Theory*, *Techniques*, *and Applications* 1^st^ edition (ed, Sone, Y.) (Brikhauser, 2007).

